# Tomato Pomace as a Valuable Resource: Characterization of Sunflower and Rapeseed Oil Extracts from Ox Heart Tomato (*Lycopersicon esculentum*)

**DOI:** 10.3390/foods14101662

**Published:** 2025-05-08

**Authors:** Dumitriţa Flaiş, Mariana Spinei, Mircea Oroian

**Affiliations:** 1 Faculty of Food Engineering, “Ştefan cel Mare” University of Suceava, 13th University Street, 720229 Suceava, Romaniamariana.spinei@fia.usv.ro (M.S.); 2 Integrated Center for Research, Development and Innovation in Advanced Materials, Nanotechnologies, and Distributed Systems for Fabrication and Control (MANSiD), “Ştefan cel Mare” University of Suceava, 13th University Street, 720229 Suceava, Romania; 3 Suceava-Botoșani Regional Innovative Bioeconomy Cluster Association, 720229 Suceava, Romania

**Keywords:** tomato pomace, extraction, oils, characterization, antioxidant activity, FTIR, HPLC

## Abstract

This study investigates the potential use of tomato pomace, a by-product of tomato pulp and seeds, as a functional additive in sunflower and rapeseed oils. The aim was twofold: to improve the oxidative stability of the oils and to enhance their nutritional value for potential food applications. Extractions were performed using different concentrations of tomato pomace (2%, 4%, and 6%) at varying temperatures (40°, 50°, and 60 °C) and stirring speeds (150, 200, and 250 rpm). The resulting samples were evaluated for antioxidant activity, peroxide value, and color characteristics. To further assess oil composition and stability, advanced analytical techniques were employed. Fourier-transform infrared spectroscopy (FTIR) identified key functional groups and structural modifications, while differential scanning calorimetry (DSC) revealed differences in thermal stability across the samples. High-performance liquid chromatography (HPLC), optimized for compound separation and detection, identified several carotenoids, including canthaxanthin, lutein, lycopene, and alpha carotene. Notable differences between extracts made with sunflower versus rapeseed oil were observed, reflecting variations in chemical composition, extraction efficiency, and oil–pomace interactions. In conclusion, the incorporation of tomato pomace into vegetable oils not only enhances oxidative and thermal stability but also enriches the oils with valuable bioactive compounds, supporting their potential use in functional food formulations.

## 1. Introduction

The tomato processing industry represents a pivotal segment within the agri-food sector, with an annual global processing volume exceeding 40 million tons of tomatoes [1]. In the contemporary era, tomatoes have firmly established themselves as one of the most widely grown and appreciated vegetables in Romania [2]. Classified as both vegetables and fruits, tomatoes are appreciated not only for their variety of colors, sizes, and shapes but especially for their sweet taste and juicy texture. In terms of chemical composition, they contain around 80% water, 2% protein, 3% sugars, along with minerals, vitamins, and organic acids such as citric, malic, and pectic acids as well as oxalates [3]. The solid residue resulting from the industrial processing of tomatoes, known as tomato pomace, consists primarily of peels and seeds. This by-product is currently utilized as animal feed, fertilizer or, in certain instances, disposed of as waste [4]. Tomato processing generates significant amounts of by-products, primarily peels and seeds, which account for 10–40% of the total processed mass [5]. Known as tomato pomace, this waste material is rich in valuable bioactive compounds such as carotenoids, phenolics, and vitamins [6,7]. Lycopene, the dominant carotenoid in ripe tomatoes, is present in concentrations up to five times higher in the peel than in the pulp [8,9]. Due to its strong antioxidant activity, lycopene has been linked to reduced risks of cardiovascular and degenerative diseases [10,11,12].

Given the human body’s inability to synthesize carotenoids, their dietary intake is essential [13]. However, their fat-soluble and hydrophobic nature complicates extraction and bioavailability, making the use of lipid-based carriers, such as vegetable oils, a suitable alternative [14,15]. Sunflower and rapeseed oils not only facilitate carotenoid solubilization but also add nutritional value to the final extract [16,17].

The recovery of carotenoids from tomato pomace supports both waste valorization and the demand for natural antioxidants in food, pharmaceutical, and cosmetic applications. Various strategies have been developed to extract these compounds, although challenges remain due to poor water solubility, oil crystallization, and the limitations of chromatographic mobile phases [15,18,19].

The objective of this study was to valorize by-products, such as the peels and seeds of ox heart tomatoes, which are currently considered waste. However, upon transformation, the pomace was revealed to be a valuable source. The extraction process utilized sunflower oil and rapeseed oil, yielding a functional oil that is enriched with carotenoids from the tomato pomace and antioxidants. These can be employed in the food industry healthy cooking oil and nutritional supplements and in various food products, including mayonnaise. The potential of this functional oil is further extended by its inherent antioxidant properties, which renders it suitable for utilization in the pharmaceutical and cosmetics industries. In the former sector, it finds application in the prevention of cardiovascular diseases, while in the latter, it contributes to skin protection and anti-aging properties. The present study aims to provide a concise overview of the available methods for the extraction of carotenoids, with a particular focus on lycopene, from tomato processing by-products. In this study, the extraction of carotenoids from the peel and seeds of ox heart tomatoes was performed with two types of vegetable oils (sunflower oil and rapeseed oil) by two methods: shaking and ultrasonication using variable temperatures (40–80 °C), shaking speeds (200–400 rpm), and different proportions of powdered peel and seeds from sunflower oil and rapeseed oil tomatoes (2.5–5.5%, gr/v). The study presents extraction techniques using two types of oils for the recovery of carotenoids from tomato pomace. Prior to this, a Box–Behnken experimental design was performed to optimize the extraction process from tomato pomace.

## 2. Materials and Methods

### 2.1. Materials

The pomace of ox heart tomatoes (*Lycopersicon esculentum*) was subjected to a drying process at a temperature of 40 °C, followed by a grinding procedure using a grinder BOSCH TSM6A013B. Although the moisture content of the dried pomace was not determined in this study, the drying process was carried out until the sample exhibited a constant weight, suggesting minimal residual moisture. The resultant product was then subjected to a sieving process that employed a series of Retsch AS200 Basic (Retsch GmbH, Haan, Germany) vibrating screens, utilizing four distinct screen sizes of <700 μm, <500 μm, <300 μm, and <200 μm, in that sequence. The product exhibiting a grain size between <200 and >300 μm was then selected for further analysis.

The following substances were procured from Sigma-Aldrich (Hamburg, Germany): chloroform, glacial acetic acid, potassium iodide, sodium thiosulfate, DPPH, distilled water, and methanol MTBE (methyl tert-butyl ether). Sunflower oil and rapeseed oil were procured from a local supermarket.

### 2.2. Methods

#### 2.2.1. The Extraction of Bioactive Compounds from Tomato Peels and Seeds by Maceration in Sunflower and Rapeseed Oil

The extraction of bioactive compounds was conducted using a Box–Behnken design (Table 1), employing sunflower oil and rapeseed oil as solvents. The experimental procedure was executed within a flask, initially filled to a volume of 25 mL with the designated type of oil. The samples were subjected to stirring at constant temperatures (40, 60, and 80 °C) and variable rotational speeds (150, 200, and 250 rpm) using a mechanical stirrer. The Box–Behnken design was applied to optimize the extraction process using both rapeseed oil and sunflower oil as extraction media, with varying tomato pomace-to-oil ratios (ROO) ranging from 2.5 to 5.5% (*w*/*v*). The selection of the Box–Behnken design (BBD) was made on the basis that it requires fewer runs in cases of three or four variables and because it is useful and practically feasible for the study [20]. Following extraction, the samples were subjected to a process of centrifugation and subsequently stored at 4 °C with the objective of preserving the liquid state and homogeneity of the oil, thereby ensuring the stability of the bioactive compounds and averting the solidification of the samples and phase separation. The extraction methods selected were agitation and ultrasonication due to their simplicity, cost effectiveness, and scalability for potential industrial applications. Ultrasonication has been demonstrated to be a particularly effective method of enhancing mass transfer and disrupting cell walls, thereby facilitating the release of intracellular carotenoids into the oil matrix. Notwithstanding the advantages inherent in both methods, it is important to acknowledge the limitations that are characteristic of each. For instance, extensive ultrasound or elevated temperatures can result in the thermal degradation of carotenoids, which are susceptible to oxidative and thermal stress. These issues were meticulously deliberated during the optimization of the extraction parameters.

#### 2.2.2. Color Determination

The color was determined on the basis that the pomace in tomatoes contains lycopene, a carotenoid that endows the tomato with its brick-red coloration. The color of extracts was determined as described by Stinco, with some modifications [21]. A volume of 5 mL from each sample was transferred to a transparent container. The CIE L*a*b* scale was used to measure the volume, and a colorimeter spectrophotometer model CM-5 (Konica Minolta, Foster City, CA, USA) was utilized to perform the necessary measurements. To ensure the reliability of the results, three replicates were considered for each sample. We can read color in coordinates L*, a*, and b as follows:

(1) $$C*=\sqrt{\left({a*}^{2}+{b*}^{2}\right)}$$

where C*—color intensity a*—the red/green coordinate in the CIE Lab color space, where positive values indicate a shift toward red, and negative values indicate a shift toward green; b*—the yellow/blue coordinate in the CIE Lab color space, where positive values indicate a shift toward yellow, and negative values indicate a shift toward blue.

#### 2.2.3. The Antioxidant Activity

To measure the antioxidant capacity of oils, a DPPH radical scavenging basis was used, following a technique described by Brand-Williams and co-workers (1995), with some modifications [22].

The test is based on the color change of the DPPH solution: The solution is initially violet and turns yellow when all free radicals are neutralized by antioxidants. Initially, a solution of oil in chloroform (10% *w*/*v*) was prepared, and 1 mL of this was added to a solution of DPPH in chloroform (4 mL, concentration 6 × 10⁻^5^ M). After this, the mixture was agitated, and the absorbance was measured. Concurrently, the control group comprised the measurement of the DPPH solution (devoid of oil). The mixture was then left to incubate for 30 min in the dark at room temperature, after which the absorbances were measured at 518 nm using a UV–vis spectrophotometer.

The antioxidant activity (AA) of the samples was calculated using Formula (2):

(2) $$AA\left(\%\right)=\frac{A_{control-}A_{sample}}{A_{control}}\;\times \;100$$

where $A_{control}$—the value of the control sample’s absorption; $A_{sample}$—the value of the samples that have undergone testing.

#### 2.2.4. Determining the Refractive Index (RI) and the Peroxide Value (PV)

The refractive indices of the samples were measured using the ABBE Leica Mark Plus refractometer (Leica Microsystems, Wetzlar, Germany). The measurements were performed at a temperature of 20 °C with an accuracy of ±1 × 10^−4^, and all 30 samples were read at room temperature. To ensure the most accurate measurements, it is essential that both the sample and the refractometer prism be clean and free from impurities. Following the addition of the requisite volume of sample, the apparatus was adjusted, and the value was then read from its optical scale. The peroxide value was determined on all the samples by taking 1 g of the product from each and adding it into another conical flask measuring 2 mL of water. To both flasks, 10 mL of chloroform was added and shaken until the fat was dissolved. Over this, 15 mL of glacial acetic acid and 1 mL of potassium iodide were added, and the flasks shaken again. The flasks were left in the dark for 5 min, then 1 mL of starch was added, and the titration continued until the yellow color disappeared. The peroxide value is expressed in milliequivalents of oxygen peroxide per kilogram, calculated by the following Formula (3):

(3) $$\mathrm{PV}=\frac{\left(V-v\right)*F*0.00127*100}{G}$$

where V = ml of 0.01 n sodium thiosulphate solution consumed when titrating the fat sample; v = ml 0.01 n sodium thiosulphate solution consumed for control; F = sodium thiosulphate solution factor; 0.00127 = amount of iodine (g) equivalent to 1 mL of 0.01 n thiosulphate solution; G = weight of fat analyzed.

#### 2.2.5. Individual Determination of Carotenoids

A comprehensive analysis of carotenoids was conducted on the extract using a high-performance liquid chromatograph (SHIMADZU, Tokyo, Japan) equipped with a diode array detector. The carotenoids analyzed included zeaxanthin, canthaxanthin, trans-beta-apo-8′-carotenal, astaxanthin, alpha-carotene, beta-carotene, lutein, and lycopene. Prior to analysis, the samples underwent saponification, after which they were analyzed. Separation was performed on a CT99S05-2546WT column, which has the following dimensions: 150 mm in length and a particle diameter of 4.6 mm with 5 μm separation. Elution was carried out using a solvent system consisting of solvent A (methanol/MTBE*/water in 81/15/4 ratio) and solvent B (methanol/MTBE*/water in 86/90 ratio) at a flow rate of 1 mL/min. The gradient was 0–100% B (0–90 min). The compounds were detected at 450 nm.

#### 2.2.6. Evaluation of the Quality of Two Types of Oils Extracted from Tomatoes with Pomace by FTIR Spectroscopy

The samples were previously lyophilized to remove moisture while preserving thermolabile compounds. The absorption spectra were then obtained using Fourier transform infrared (FTIR) spectroscopy (Nicolet iS20, Thermo Scientific Inc., Waltham, MA, USA), equipped with an attenuated total reflectance (ATR) accessory. The lyophilization step ensured sample stability and minimized interference during spectral acquisition. The samples were then placed in contact with the ZnSe crystal by applying a loading pressure, and the spectra were scanned in a range from 550 cm^−1^ to 4000 cm^−1^ at room temperature. The results were analyzed with Spectrum ^TM^ 10 software (Thermo Fisher Scientific Inc., Waltham, MA, USA).

#### 2.2.7. DSC (Differential Scanning Calorimetry) Analysis

Calorimetric analyses were performed using a TA4000 differential scanning calorimeter (Mettler-Toledo, Greifensee, Switzerland) connected to a GraphWare TAT72.2/5 software (Mettler-Toledo, Greifensee, Switzerland). Heat flux calibration was performed using indium (heat of fusion 28.45 J/g). Temperature calibration was carried out using hexane (p.t. −93.5 °C), water (p.t. 0.0 °C), and indium (p.t. 156.6 °C). Samples were prepared by carefully weighing 10–15 mg of material into 20 L aluminum DSC containers, which were then closed without hermetic sealing. An empty container was used as a reference, samples were heated under nitrogen flow (10 mL/min) at 40 °C for 15 min to erase the crystallization memory, and they were cooled to −80 °C and then heated from −80 °C to 20 °C. It should be noted that heating does not alter the oxidation level of the samples, and the scanning speed was 2 °C/min. The beginning and the end of the melting transition were designated as on-set (Ton) and offset (Toff) transition points, respectively; i.e., the points where the extrapolated baseline intersects the extrapolated slope of the curve. Tpeak corresponds to the maximum peak temperature. The results were normalized to account for weight variation of the samples. The total peak enthalpy of crystallization (DHcr) and melting (DHm) was obtained by integration. The program STAR ever, 8.10 (Mettler-Toledo, Greifensee, Switzerland) was used to plot and analyze the thermal data.

## 3. Results and Discussion

### 3.1. Influence of Temperature on Colour in CIE L*a*b** Coordinates

The color parameters L*, a*, and b* have been extensively utilized to describe and communicate colors [23]. As illustrated in Table 2, the L*a*b* values for sunflower oil enriched with tomato extracts demonstrate the influence of these parameters on color perception. The data obtained demonstrate that the solid-to-liquid (S/L) ratio exerts a significant influence on the physico-chemical characteristics of the finished product. It is evident that as the S/L ratio decreases, the color intensity increases, and conversely, as the S/L ratio increases, the color intensity decreases. Additionally, it is noteworthy that temperature constitutes an additional factor influencing color, as extractability increases in proportion to rising temperature.

As demonstrated in Table 2, the L* value ranges from 91.34 to 97.01, indicating a spectrum of light shades from lighter (in the case of pure oil) to darker (with the addition of tomato powder and modification of extraction conditions). The a* values are negative, suggesting a green tint, influenced by the pigment composition and the amount of tomato powder added. The lowest value of −6.35 occurs in samples with greater tomato powder content. b* values vary considerably, from 33.53 to 87.56, indicating a wide range of yellows depending on processing conditions. C* ranges from 3 3.97 to 87.59, which reflects the degree of color saturation (with intensification of color as tomato powder is added and the processing parameters changed, with small fluctuations depending on processing parameters.

The color difference (ΔE*) values varied considerably, ranging from 0.00 for the pure oil (used as a reference) to 32.66 in the sample extracted with 6% tomato powder at 60 °C and 250 rpm, as shown in Table 2. This wide variation indicates substantial changes in the color of the oil because of both the incorporation of tomato pomace and the influence of processing parameters such as temperature and time. A higher ΔE* value reflects a more pronounced color shift compared to the pure oil, suggesting that pigment transfer, particularly of carotenoids, and possible oxidative reactions significantly altered the visual characteristics of the oil. These results, detailed in Table 3, underline the impact of matrix composition and process conditions on the final appearance of the oil. The results of the samples extracted with rapeseed oil are shown in Table 4. The lightness (L*) values range from 87.90 to 91.30, indicating lower lightness compared to sunflower oil. The brightness of the samples is influenced by temperature and the addition of tomato powder. The a* values are also negative, indicating green shades, with a maximum point of −0.57 in the case of the rapeseed oil sample. The b* values are much higher compared to a*, reflecting a more pronounced yellowing (from 131.02 to 133.66). The C* values are higher in comparison to sunflower oil, with the values for rapeseed oil range from 131.02 to 133.66, suggesting an intensification of the yellow color. The h values, ranging from 88.20 to 93.08, indicate a predominance of yellow-green. The ΔE values, ranging from 31.99 to 40.88, demonstrate significant color changes during the process. A few differences can also be observed between samples extracted with sunflower oil and those extracted with rapeseed oil. The L* values indicate that sunflower oil appears brighter and lighter in color compared to rapeseed oil. In contrast, the b* values reveal that rapeseed oil exhibits a more intense yellow hue, whereas sunflower oil shows a less pronounced yellow coloration. This is further supported by the higher C* values observed in rapeseed oil, which reflect greater color intensity and saturation. Although both oils display negative a* values, indicating a tendency toward green, rapeseed oil shows greater variability in this parameter. Overall, these differences result in a more saturated, yellowish appearance for rapeseed oil, while sunflower oil remains lighter and less saturated in tone.

Yovcheva et al. [24] observed that the L* value decreased with the incorporation of extra virgin olive oil, while the levels of β-carotene and chlorophyll increased. The influence of the green component was found to increase in proportion to the increase in olive oil content. A linear relationship was identified between b* values and olive oil concentration. The greatest color difference was observed between pure olive oil and sunflower oil. Romanić et al. [25] analyzed the color parameters of oil blends obtained by combining sunflower oil with linseed oil. In terms of color difference, the blended oils showed significantly higher ΔE values as the proportion of linseed oil increased. To illustrate this point, consider the blend of 80% sunflower oil (S) and 20% linseed oil (F), which exhibited a ΔE* of 7.02 ± 0.01. In contrast, the blend containing 100% sunflower oil (S) and 0% linseed oil [25] showed a significantly lower ΔE of 1.28 ± 0.02.

In the analysis conducted by Giacomelli et al. [26], the mixtures of sesame oil and rapeseed oil exhibited a high and almost constant brightness. The parameter a* of the blends ranged from 0 to 5, indicating a greenish hue in the oil. The b* value of the blends was found to be contingent on the concentration ratio of sesame oil and rapeseed oil in the blends. Pure rapeseed oil exhibited the highest b* value of 88, corresponding to the yellow color, which may be attributed to the presence of carotenoids. In contrast, sesame oil demonstrated the lowest b* value of 15, defining a pale-yellow color, which is likely associated with a reduced concentration of carotenoids.

### 3.2. Antioxidant Activity (DPPH) Assay and Total Lycopene Quantification

This section examines the lycopene values (mg/100 g) and antioxidant activity (percentage DPPH radical scavenging) of two types of oils: sunflower and rapeseed enriched with tomato pomace (Table 4). The lycopene results demonstrate that sunflower oil typically contains a higher amount of lycopene compared to rapeseed oil. For instance, the first sample exhibited 1.67 mg/100 g of lycopene in sunflower oil, while rapeseed oil contained only 1.21 mg/100 g. This observation suggests a higher concentration of lycopene in sunflower oil, which is a significant antioxidant. Furthermore, sunflower oil demonstrated higher antioxidant activity (DPPH) compared to rapeseed oil. For instance, 63.58% of DPPH radicals were scavenged by sunflower oil, while for rapeseed oil, the figure was only 50.35%. However, this pattern was not consistent across all samples; for sample 6, rapeseed oil exhibited higher antioxidant activity (67.38%) compared to sunflower oil (57.43%).

Overall, sunflower oils were found to exhibit higher antioxidant activity, a finding that may be associated with higher lycopene concentrations. However, it should be noted that antioxidant activity exhibited significant variation among the samples, suggesting the potential influence of other variables, such as the extraction process, on the observed outcomes.

The DPPH assay is a well-established method for evaluating the free radical scavenging capacity and antioxidant activity of diverse samples. In this assay, the DPPH solution, which exhibits a violet color, is reduced by antioxidants (e.g., phenolic compounds and flavonoids), resulting in a color change from violet to yellow, thereby indicating antioxidant activity [27]. As stated by [28], the antioxidant activity of sunflower oils (SFO) was determined by DPPH assay, with the resultant readings of the absorbances being measured at 492 nm. The investigation revealed that the oil samples exhibited significantly divergent free radical scavenging activities, with the crude oil demonstrating the highest antioxidant activity of 55.64%, while the deodorized oil exhibited the lowest antioxidant activity of 35.87%, at a concentration of 50,000 µg/mL ethanolic solution [28].

### 3.3. Analysis of Refractive Index and Peroxide Value

The resulting values fall within the range of 1.4702–1.4712 (Table 5 and Table 6), indicating that the oils are within the range specified by Capocasale et al. [29]. Sunflower oil exhibited the lowest value, while rapeseed oil demonstrated slightly higher results, though the differences were not significant. Numerous factors can influence the refractive index of these two oils. The temperature at which the refractive index is measured can be a factor; therefore, it is essential to maintain the temperature found during the reading. The chemical composition of the oils can also influence the refractive index, as oils contain different chemical compounds. Finally, the presence of pomace, a by-product of tomato extraction, has been identified as a factor capable of altering the refractive indices of the oils [30]. The titration method was selected for determining the peroxide value due to its practicality and accessibility. The findings of the present study demonstrate that the highest peroxide value was observed in sample 15, which was extracted at 80 °C, 200 rpm, and 3 g pomace using rapeseed oil: 6.14 meq/kg, and the lowest amount was found in sample 3 extracted with sunflower oil at 40 °C, 200 rpm, and 1 g of tomato pomace, having a value of 0.001. The findings of the research [31] revealed that crude tomato seed oil exhibited the lowest peroxide value, while the highest value of 8.14 was observed for the mixture of tomato seed oil and sunflower oil. Among the three types of oil analyzed—tomato seed oil, sunflower oil, and blended oil—it was found that the blend retained the natural antioxidants the best, thus having the lowest peroxide value [31]. According to studies conducted on chia oil, the refractive index was 1.4761 but at 25 °C [32]. However, this value was higher than those reported by Codex Stan 210 [33] for sunflower (1.461), safflower (1.467), and soybean (1.466) oils at 40 °C. The research of [23] demonstrated that the refractive index is influenced by both the analysis temperature and the unsaturated fatty acid content. It was determined by the researchers that higher analysis temperatures resulted in lower refractive index values and that higher unsaturated fatty acid content was associated with higher refractive index values [34]. Edible oils are susceptible to oxidation during processing and storage, and this process can have a detrimental effect on both oil quality and consumer health. The measurement of peroxide value is crucial, as it is one of the most important indicators used to assess the degree of lipid oxidation and to determine the quality of the oil [30].

### 3.4. Model Fitting of Antioxidant Activity, Oxidative Stability, and Color Parameters

A Box–Behnken design was employed to optimize the yield of lycopene extraction using maceration. The results are summarized in Table 7. The experimental design yielded a total of 15 batch runs [35]. The maceration in the two types of oils was carried out because a comparison was also desired to determine which of the two offers a more efficient solubilization of lycopene and stability of the extract.

The Box–Behnken design relied on the second-order (quadratic) polynomial response surface model using the following equation:

(4) $$y=b_{o}+\sum_{i=1}^{n}(b_{i}x_{i})+\sum_{i=1}^{n}(b_{ii}x_{ii}^{2})+\sum_{ij=1}^{n}\;(b_{ij}x_{i}x_{j})$$

where y stands for the predicted response (IP), xi represents the coded levels of the design variable (ratio, temperature, and time), b_0_ is a constant, bi represents the linear effects, bii denotes the quadratic effects, and bij represents the interaction effects.

The second-order polynomial equations in coded form for sunflower oil extraction are presented below.

Lycopene (mg/100 g) = 1.63 − 0.006 ·$X_{1}$− 0.04${*X}_{2}$ + 0.13 ·$X_{3}$ + 0.01 ·$X_{1}\cdot X_{2}$ + 0.002 ·$X_{1}\cdot X_{3}$ + 0.06 ·$X_{2}\cdot X_{3}$− 0.14 ·$X_{1}^{2}$+ 0.07 ·$X_{2}^{2}$+ 0.15 ·$X_{3}^{2}$

DPPH (%) = 52.61 − 4.53 · $X_{1}$ + 1.28 · $X_{2}$ + 2.07 · $X_{3}$+ 0.51 ·$X_{1}\cdot X_{2}$ − 6.46 ·$X_{1}\cdot X_{3}$ + 0.77 ·$X_{2}\cdot X_{3}$+ 0.74 ·$X_{1}^{2}$ + 3.82 ·$X_{2}^{2}+$ 4.13 ·$X_{3}^{2}$

IP (meq O**_2_**/kg) = 15.05 − 0.03 · $X_{1}$ − 1.87 · $X_{2}$ + 0.33 · $X_{3}$+ 2.92 ·$X_{1}\cdot X_{2}$ − 3.25 ·$X_{1}\cdot X_{3}$ − 0.17 ·$X_{2}\cdot X_{3}$− 1.37 ·$X_{1}^{2}$ − 0.65 ·$X_{2}^{2}$+ 0.82 ·$X_{3}^{2}$

Color difference (ΔE*) = 32.61 + 2.11 · $X_{1}$ + 3.95 · $X_{2}$ − 2.96 · $X_{3}$− 1.24 ·$X_{1}\cdot X_{2}$ − 1.37 ·$X_{1}\cdot X_{3}$ + 0.49 ·$X_{2}\cdot X_{3}$− 8.25 ·$X_{1}^{2}$ − 10.59 ·$X_{2}^{2}-$9.23 ·$X_{3}^{2}$

The second-order polynomial equations in coded form for rapeseed oil extraction are presented below.

Lycopene (mg/100 g) = 1.17 + 0.04 · $X_{1}$ +0.005 · $X_{2}$ + 0.001 · $X_{3}-$0.022 ·$X_{1}\cdot X_{2}$ + 0.07 ·$X_{1}\cdot X_{3}$ + 0.14 ·$X_{2}\cdot X_{3}$− 0.07 ·$X_{1}^{2}$ + 0.1325 ·$X_{2}^{2}-$0.19 ·$X_{3}^{2}$

DPPH (%) = 59.92 + 1.12 · $X_{1}$ + 1.64 · $X_{2}$ − 4.69 · $X_{3}$+ 1.10 ·$X_{1}\cdot X_{2}$ − 5.61 ·$X_{1}\cdot X_{3}$ − 2.18 ·$X_{2}\cdot X_{3}$− 6.51 ·$X_{1}^{2}$ − 0.82 ·$X_{2}^{2}+$ 0.30 ·$X_{3}^{2}$

IP (meq O**_2_**/kg) = 43.46 + 0.27 · $X_{1}$ + 3.42 · $X_{2}$ − 9.22 · $X_{3}$− 9.37 ·$X_{1}\cdot X_{2}$ + 10.72 ·$X_{1}\cdot X_{3}$ − 12.57 ·$X_{2}\cdot X_{3}$− 9.01 ·$X_{1}^{2}$ − 6.91 ·$X_{2}^{2}-$ 4.91 ·$X_{3}^{2}$

Color difference (ΔE) = 33.42 + 0.89 · $X_{1}$ − 0.27 · $X_{2}$ − 0.22 · $X_{3}$+ 0.71 ·$X_{1}\cdot X_{2}$ − 0.67 ·$X_{1}\cdot X_{3}$ − 0.32 ·$X_{2}\cdot X_{3}$− 0.76 ·$X_{1}^{2}$ − 0.79 ·$X_{2}^{2}-$0.9 ·$X_{3}^{2}$

The model is significant in most cases, especially in the context of sunflower oil, as it demonstrates that the included factors adequately explain the variation in the analyzed responses. Conversely, for rapeseed oil, the significance of the models is comparatively weaker in certain instances, yet they remain beneficial for general interpretations. Factors X_2_ and X_3_ as well as interactions X_13_ and X_12_ frequently appear significant, indicating an important influence on the responses. The R^2^ and adjusted R^2^ values are generally high (above 0.70) for sunflower oil, indicating a good fit. For rapeseed, the fit is more moderate but acceptable. Adequate precision (Adeq Precision) is above the recommended threshold (>4) in all cases, indicating an acceptable signal-to-noise ratio. The coefficient of variation (CV%) is minimal in most cases (below 10–15%), suggesting good reproducibility of the data.

In the context of PV, the model demonstrates significance for samples extracted with sunflower oil, with the solid-to-liquid ratio (X_2_) identified as a substantial factor (*p* < 0.05). Conversely, for rapeseed oil, the model does not exhibit significance. About lycopene, the model was found to be non-significant for both extraction solvents. However, some individual terms demonstrate borderline significance in the sunflower oil samples. In the context of DPPH, the model attained significance for sunflower oil, with X_1_, X_3_, and X_13_ identified as significant terms (*p* < 0.05). Conversely, for rapeseed oil, the model was found to be non-significant. In the context of color variation, both models demonstrate significance, though the rapeseed oil model exhibits a more pronounced effect. It is notable that several quadratic and interaction terms (X_13_, X_12_, and X_23_) are significant, particularly within the rapeseed oil model. In general, models based on sunflower oil demonstrate higher levels of significance and superior statistical fit (R^2^, Adeq Precision) in comparison to those based on rapeseed oil.

#### Surface Response 3D Plots

**Figure 1:**
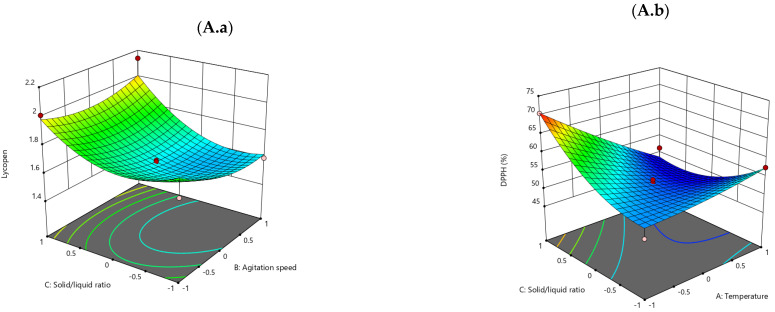

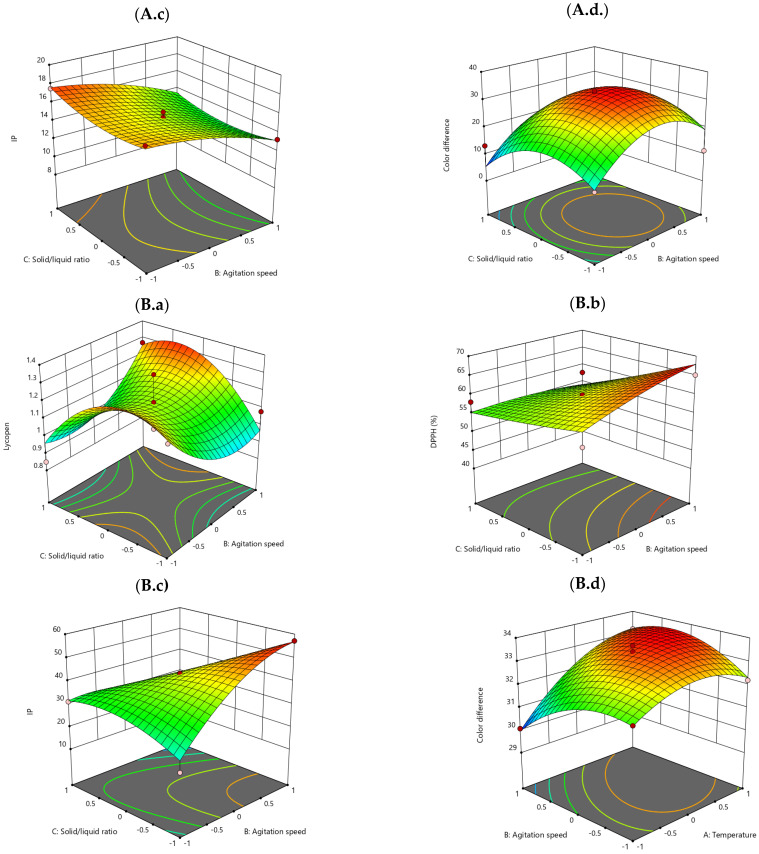
Surface Response 3D Plots: (**A.a**) Influence of solid/liquid ratio on the agitation speed of sunflower oil. (**A.b**) Influence of solid/liquid ratio on the temperature of sunflower oil. (**A.c**) Influence of the solid/liquid ratio on the agitation speed of sunflower oil. (**A.d**) Influence of solid/liquid ratio on the agitation speed of sunflower oil. (**B.a**) Influence of the solid/liquid ratio on the agitation speed of rapeseed oil. (**B.b**) Influence of the solid/liquid ratio on the agitation speed of rapeseed oil. (**B.c**) Influence of the solid/liquid ratio on the agitation speed of rapeseed oil. (**B.d**) Influence of the agitation speed on the temperature rapeseed oil.

As illustrated in Figure 1(Aa), the effect of the solid-to-liquid ratio (C) and agitation speed (B) on lycopene content in sunflower oil is demonstrated. A parabolic trend is observed, indicating that lycopene content is higher at the extremes of both variables, suggesting potential optimal conditions at the margins of the tested range. From Figure 1(Ab) (Effect of solid-to-liquid ratio (C) and temperature (A) on DPPH (%)—antioxidant activity), it is evident from the data that DPPH values increase with an elevated solid-to-liquid ratio and a reduced temperature. This finding suggests that the conditions for extracting antioxidant compounds are favorable within these parameters. In Figure 1(Ac) (Effect of solid-to-liquid ratio (C) and agitation speed (B) on PV (induction period)), higher PV values are observed at higher solid-to-liquid ratios and lower agitation speeds. This finding suggests that improved oxidative stability is achieved under milder agitation conditions In Figure 1(Ad) (Effect of solid-to-liquid ratio (C) and agitation speed (B) on color difference (ΔE)), it is evident from the central region of the plot that color differences increase. Minimum ΔE values are observed at lower values of C and B, indicating less visual impact under gentler extraction conditions.

In Figure 1B (Rapeseed oil) and Figure 1(Ba) (Effect of solid-to-liquid ratio (C) and agitation speed (B) on lycopene content), the analysis indicates a substantial increase in lycopene content in regions characterized by elevated agitation speeds and moderate-to-low solid-to-liquid ratios. This finding suggests a synergistic effect between these two variables. Figure 1(Bb) (Effect of solid-to-liquid ratio (C) and agitation speed (B) on DPPH (%)) shows a clear linear increase in DPPH is observed with both variables. This suggests that under more intense process conditions, antioxidant extraction is more efficient. In Figure 1(Bc) (Effect of solid-to-liquid ratio (C) and agitation speed (B) on PV), it can be seen that PV values significantly increase under conditions of high agitation and a high solid-to-liquid ratio. These parameters appear to enhance oxidative stability. Figure 1(Bd) (Effect of agitation speed (B) and temperature (A) on color difference (ΔE)) shows that color differences increase with both higher temperature and agitation speed, likely due to a combination of thermal and mechanical effects.

Sunflower oil demonstrates a heightened sensitivity to alterations in the solid-to-liquid ratio, as evidenced by variations in color difference. In contrast, rapeseed oil demonstrates a more pronounced response to variations in DPPH under different agitation speeds, suggesting divergent dynamics in antioxidant extraction. The obtained plots suggest clear optimal zones for maximizing lycopene content, DPPH activity, and PV while minimizing color difference, depending on the oil type and process goals. The regression models used are effective in predicting the outcomes, as evidenced by the parabolic and planar shapes of the response surfaces. This indicates that the models can predict well across the tested design space.

### 3.5. Quantification of Carotenoids in Oil Extracts Using HPLC-DAD Analysis

**Figure 2:**
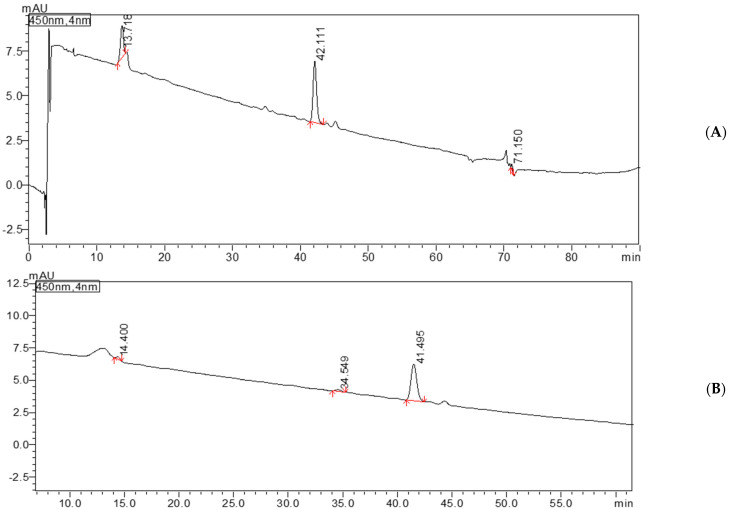
HPLC-DAD chromatogram at 450 nm for the standard (100 mg/L); for sample (**A**) (extracted with rapeseed oil at 40 °C, 200 rpm, 6 g tomato pomace): canthaxanthin—peak 1 (minute 13.718), lutein—peak 2 (minute 42.111), lycopene—peak 3 (minute 71.15) (**B**) (extracted with sunflower oil at 60 °C, 200 rpm, 4 g tomato pomace): canthaxanthin—peak 1 (minute 14.4), alpha carotene—peak 2 (minute 34.549), and lutein—peak 3 (minute 41.495).

Quantification of carotenoids in the extracts studied was performed by HPLC-DAD analysis based on calibration curves constructed using standard solutions of β-carotene, lutein, and zeaxanthin dipalmitate. In Figure 2 is presented a typical chromatorgram of standards and of a sample. Carotenoids are also concentrated by an industrial process called fractionation.

This study investigated carotenoids in two types of oil—rapeseed oil and sunflower oil—using the HPLC-DAD method, which allows carotenoids to be analyzed at a wavelength of 450 nm. The aim was to identify and quantify the carotenoids in these oils based on the retention times and peak areas obtained from the chromatographic analysis. This paper shows the data obtained from the determination of carotenoids in a sample of sunflower oil using HPLC-DAD. This allows the identification and quantification of different types of carotenoids in a sample based on the retention time (RT) and signal area for each carotenoid. In this study, sunflower oil and rapeseed oil, both with the same parameters (80 °C, 150 rpm, and 2 g pomace of tomatoes), were used for the HPLC measurements.

In the first sample extracted with rapeseed oil, three main carotenoids were observed. Cantaxanthin was detected with a retention time of 13.71 min. This is a carotenoid found in both rapeseed oil and sunflower oil but with a slightly different retention time. Lutein was identified with a retention time of 42.11 min, and lycopene appeared with a retention time of 71.15 min. In the second sample, extracted with sunflower oil, the presence of carotenoids was again observed but with different retention times compared to rapeseed oil. Cantaxanthin was observed at minute 14.4, a slightly longer retention time compared to rapeseed oil. Lutein was detected at minute 41.49, and another carotenoid, i.e., α-carotene, appeared at minute 34.54, which did not occur in rapeseed oil.

These results suggest that although some carotenoids are present in both types of oil, such as canthaxanthin and lutein, the retention times of the different carotenoids may indicate differences in their chemical behavior within the two oils. Rapeseed oil and sunflower oil contain different profiles of carotenoids, which may indicate different interactions between the carotenoids and the chemical components of the oil. In addition, sunflower oil contains α-carotene, and rapeseed oil contains lycopene, carotenoids that are not found in either oil.

As carotenoids are likely to increase in importance and value, the recovery of carotenoids from palm oil and palm oil by-products is important. In addition, some studies are being carried out to recover and concentrate carotenoids from different sources of palm oil to obtain a high carotenoid content palm oil for pharmaceutical use [36].

Column chromatography has been used in previous studies using different adsorbents, and reversed-phase HPLC has been shown to have several advantages for the separation of carotenoids in oil. According to studies conducted by [37], the major carotenoids present in crude palm oil are α- and β-carotene, which account for about 80–90% of the total carotenoid content. Other carotenoids are present as minor components, including -carotene, phytofluene, phytoene, lycopene, neurospores, 7-carotene, and α-carotene [37].

In the study conducted by Corbu et al. [38], it was observed that the carotenoid content varies according to the type of oil used. Extra virgin olive oil contains the most carotenoids (21.5 mg/kg), followed by extra virgin sunflower oil (6.3 mg/kg) and refined sunflower oil (4.8 mg/kg). After the ultrasonic extraction process, the carotenoid content increases significantly, reaching 157.84 mg/kg in extra virgin olive oil and 137.83 mg/kg in sunflower oil. Another study reported 63.84 mg/L of carotenoids in a sea buckthorn by-product extract added to sunflower oil [38].

### 3.6. FTIR Analysis of Functional Groups in Oil Extracts

**Figure 3:**
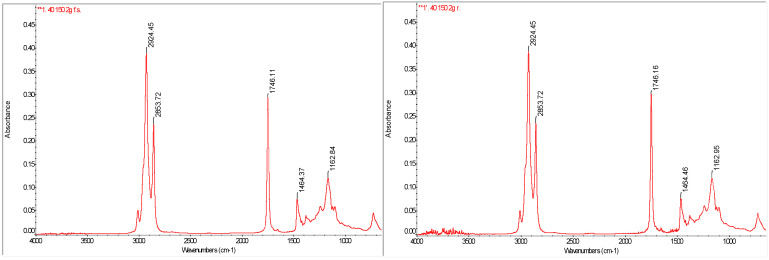
FTIR spectra for sunflower and rapeseed oil.

FTIR was used to identify and analyze the functional groups present in edible oils, highlighting the vibrational mode of specific absorption bands. The spectra are presented in Figure 3. Following spectral analysis, several characteristic functional groups were identified. The absorption band at 2924 cm^−1^ corresponds to the stretching vibrations of C–H bonds in –CH**_2_** and –CH_3_ groups, which are part of the hydrocarbon chains of fatty acids and are characteristic of lipid structures. Steric group (-C=O) was present in two distinct bands: 2678 cm^−1^, which is attributed to the stretching vibration of the carbon-oxygen bond (C=O) in the ester structure present in triglycerides, and 1743 cm^−1^, which is a strong band specific to the stretching vibrations of the carbonyl group in fatty acid esters, confirming the presence of triglycerides.

For methylene and methyl groups (-CH**_2_**-CH_3_), the absorption band at 1465 cm^−1^ is attributed to stretching vibrations of the C-H bond and is characteristic of vegetable oils. For the carbon–oxygen (C-O) bond and methylene group (-CH**_2_**), the band identified at 1155 cm^−1^ is specific to stretching vibrations of C-O bonds in the ester structure and helps to confirm the triglyceride composition of the oil. Using FTIR analysis, we were able to highlight the chemical structure of the oils tested and their stability, confirming the presence of fatty acids and triglycerides in the composition of the samples analyzed. This information is essential for characterizing the quality and oxidative stability of edible oils, providing a detailed insight into the structural changes that occur during processing and storage.

The study Poiana et al. [39] analyzed the FTIR spectra of oil samples, including olive oil (OO), sunflower oil (SFO), and the mixture of the two (OO + 50 SFO), at different stages of heat treatment (unhardened and heat-treated for 8 and 16 h). They observed absorptions in the range 3100–2800 cm^−1^. In this range, absorption bands were observed characteristic of symmetrical and non-symmetrical vibrations of CH_3_ and CH_2_ groups in the alkyl residues of the triglycerides. The absorbance at 3010 cm^−1^ was used to assess the degree of unsaturation of the oils, and the intensity of this band increased with increasing linoleic acid content in the oils; increasing the percentage of SFO in the OO + SFO mixture led to an intensification of the absorbance at 3011 cm^−1^, which allowed the stability of a strong positive correlation (R = 0.98) between the absorbance intensity and the percentage of SFO added to OO.

### 3.7. Results of DSC Analysis of Sunflower Oil

**Figure 4:**
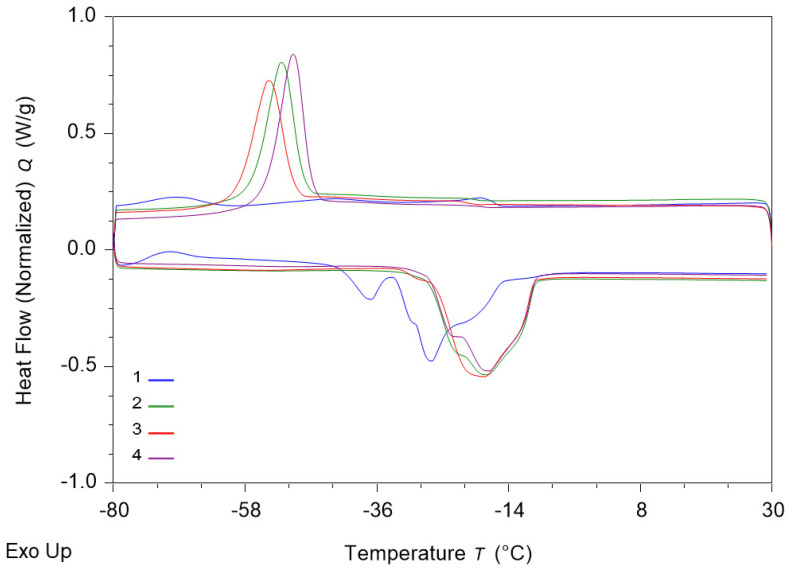
DSC thermograms of cooling and heating of sunflower oil obtained at a scan rate of 2 °C/min from 20 to −80 °C and back at T_on_ = −51.1 ± 0.2 °C, corresponding to the phase transition of the very unsaturated oily fraction with low melting point. During heating, the DSC curve shows an endothermic peak at T_on_ = −37.8 ± 0.3 °C, followed by an exothermic peak (T_on_ = −31.5 ± 0.2 °C). Blue—sunflower oil; green—Rapeseed oil; red—sunflower oil with powder; purple—rapeseed oil with powder.

A DSC thermal analysis of sunflower oil was carried out to observe its behavior during cooling and heating, using a scan rate of 2 °C/min over the time interval (Figure 4).

During cooling, at −51.1 °C ± 0.2 °C, sunflower oil showed a behavior characteristic of unsaturated oil fractions with a low melting point. This means that the unsaturated fractions of the oil solidified at low temperature, transforming into a crystalline or amorphous phase. These fractions consist of short-chain unsaturated fatty acids typical of vegetable oils.

During the heating process, an endothermic peak was observed at about −37.8 °C ± 0.3 °C. This indicates that the solidified sunflower oil fractions absorbed heat energy to transform from a solid to a liquid state. This peak reflects the melting of the unsaturated fatty acid fractions in sunflower oil, which have a molecular structure that allows this phase change.

Subsequently, at an approximate temperature of −31.5 °C ± 0.2 °C, an exothermic peak was recorded, signifying a crystallization or solidification process of oil fractions that had previously melted. This is a characteristic thermal behavior in which heat release occurs as the oil transitions from an initial state to an oxidized state. Overall, the thermal behavior of sunflower oil is characterized by low transition points, suggesting a lower thermal stability compared to saturated oils. The susceptibility of the unsaturated fractions in sunflower oil to phase changes may have ramifications for its applicability in high-temperature fabrication processes. These thermal behaviors are of significance to the food industry, in which the thermal stability of oils plays a pivotal role in manufacturing and processing processes. Oils with higher melting points exhibit greater thermal instability, leading to faster degradation when exposed to high temperatures. This can result in the retention of nutrients, the formation of toxic compounds, and a decline in the organoleptic properties of the final product. Conversely, oils with superior thermal stability are favored for their application in food processing due to their enhanced resistance to oxidation and physico-chemical changes that can compromise product quality.

The thermal stability of oils has a direct impact on the shelf life of food products as well as their safety, quality, storage, and use. Therefore, knowledge of the thermal behavior of oils can guide manufacturers in choosing the most appropriate oils for different industrial applications. Furthermore, DSC analysis can be used to assess the effects of various heat treatment processes on oils, allowing the adjustment of processing conditions to maximize the quality of the final product. Consequently, these data are imperative for the optimization of manufacturing processes, ensuring the production of high-quality food products characterized by adequate thermal stability.

The present study investigates the impact of the extraction method (sunflower oil and rapeseed oil extraction) on the thermal behavior of tomato pomace. Differential scanning calorimetry (DSC) was utilized to conduct the experiment, with measurements conducted between 30 °C and −80 °C at a heating rate of 2 °C/min. As illustrated in Figure 4, significant alterations were detected in the cooling and heating phases of sunflower oil, rapeseed oil, and tomato pomace extracted with both types of oil. The thermograms exhibited exothermic and endothermic peaks around temperatures ranging from −50 °C to 30 °C, indicating a thermal transition of the material analogous to that of beeswax or rice bran [40].

As demonstrated by the thermograms, the crystallization process exhibited a maximum at temperatures ranging from −58 °C to −36 °C, accompanied by a substantial negative heat flux. In contrast, the findings of Van Wetten et al. [41] yielded divergent results for both extra virgin olive oil and sunflower oil, with peaks ranging from −34.2 °C to −39.5 °C and −40.1 °C to −43.9 °C, respectively. The observed outcomes can be ascribed to the cooling process of triacylglycerols or to the chemical structure, which varies depending on its nature and the type of fatty acids it contains [42]. Miyagawa et al. [42] observed several crystallization phases in mixtures of rapeseed and soybean oil, with the first exothermic peak occurring between −66 °C and −53 °C and a subsequent peak between −43 °C and −40 °C. These peaks were found to be analogous to the one observed in the thermogram in Figure 4.

In the heating phase, an endothermic peak was obtained between −36 °C and −14 °C, while Van Wetten et al. [41] obtained a melting peak around −20 °C. These results indicate a different triacylglycerol composition or chemical structure, which varies depending on its nature; i.e., they have a lower melting point.

Furthermore, Miyagawa et al. [42] demonstrated that this phase is realized by three distinct endothermic peaks, with a lower bound at −39 °C and an upper bound at −48 °C for oils containing higher levels of rapeseed oil, between −32 °C and −36 °C for the phase transition in soybean oil, and between −19 °C and −17 °C for the melting of trilinolein crystals [42].

## 4. Conclusions

In conclusion, the integrated use of peroxide value, color analysis, HPLC-DAD, DSC, and FTIR techniques enabled a detailed evaluation of the physicochemical characteristics of sunflower and rapeseed oils enriched with carotenoids extracted from tomato pomace. Sunflower oil showed superior oxidative and thermal stability, as evidenced by lower peroxide values and more consistent color parameters following thermal treatment. The thermal transitions observed through DSC confirmed its structural resilience, making it especially suitable for high-temperature food processing such as frying or baking.

On the other hand, rapeseed oil exhibited a higher carotenoid content—particularly canthaxanthin and lutein—identified through the optimized HPLC-DAD method. The refinement of chromatographic conditions, including mobile phase composition, gradient profile, wavelength, flow rate, and column temperature, is critical for achieving optimal separation and sensitivity. These findings emphasize the nutritional advantage of rapeseed oil in applications where bioactive compound content is prioritized, such as in functional foods or dietary supplements.

Although rapeseed oil was more prone to peroxide formation, likely due to its higher degree of unsaturation, this is balanced by its richer bioactive profile. FTIR analysis supported these observations by identifying functional groups characteristic of fatty acids and triglycerides and indicating molecular changes related to oxidation and processing. Together, these results confirm the presence of valuable phytochemicals in both oils and offer insights into their behavior during storage and use.

Overall, this study highlights the complementary strengths of both sunflower and rapeseed oils. Sunflower oil is more suitable for thermal stability and processing performance, while rapeseed oil provides higher nutritional value through increased carotenoid content. These insights are essential for guiding the selection of oil types based on specific industrial applications and for supporting the development of healthier, functional food products derived from agro-industrial by-products.

## Figures and Tables

**Table 1:** Actual and coded design values.

Independent Variables	Coded Values		
	−1	0	1
Solvent/liquid ratio (g/mL)—X_1_	2	4	6
Temperature (°C)—X_2_	40	60	80
Stirring speed (rpm)	150	200	250

**Table 2:** Color in L*, a*, b, C*, and ΔE* coordinates of sunflower oil samples.

No.	Samples Sunflowers Oil			L*	a*	b*	C*	ΔE*
	Temperature (°C)	Agitation Speed (rpm)	Quantity—Tomato Pomace (g)					
1.	40	150	2	94.41	−5.83	53.39	59.48	5.10
2.	40	250	2	94.38	−5.44	62.05	62.29	21.86
3.	40	200	1	97.01	−5.44	33.53	33.97	12.94
4.	40	200	3	92.93	−3.91	75.50	75.60	27.64
5.	60	150	1	96.45	−5.95	39.89	40.33	8.77
6.	60	150	3	92.07	−3.35	83.63	83.70	30.44
7.	80	150	2	95.24	−5.83	53.07	53.39	17.62
8.	60	200	2	93.93	−5.37	66.08	66.29	23.73
9.	60	200	2	93.86	−5.43	66.86	67.08	24.00
10.	60	200	2	93.88	−5.44	66.51	66.73	23.92
11.	80	250	2	93.67	−5.58	68.37	68.60	24.69
12.	60	150	1	96.45	−6.05	41.68	42.11	8.71
13.	60	250	3	91.75	−2.48	87.56	87.59	31.48
14.	80	200	1	96.19	−6.35	42.83	43.29	11.05
15.	80	200	3	91.34	−2.36	88.82	88.85	32.66

**Table 3:** Color (in coordinates L*, a*, b, C*, and ΔE*) for rapeseed oil samples.

No.	Samples Rapeseed Oil			L*	a*	b*	C*	ΔE*
	Temperature (°C)	Agitation Speed (rpm)	Quantity—Tomato Pomace (g)					
1.	40	150	2	89.92	0.52	132.79	132.79	31.99
2.	40	250	2	89.91	0.83	132.95	132.95	36.48
3.	40	200	1	91.19	−2.69	131.29	131.31	33.05
4.	40	200	3	89.14	2.57	133.54	133.56	38.24
5.	60	150	1	91.30	−3.00	131.17	131.20	32.72
6.	60	150	3	88.61	3.27	133.39	133.43	39.40
7.	80	150	2	90.40	−0.57	132.42	132.42	35.25
8.	60	200	2	89.76	0.80	132.77	132.77	36.84
9.	60	200	2	89.70	0.95	132.88	132.88	36.99
10.	60	200	2	89.77	0.80	132.92	132.92	36.82
11.	80	250	2	89.56	0.93	132.45	132.45	37.32
12.	60	150	1	91.07	−2.17	131.62	131.64	33.41
13.	60	250	3	88.35	4.13	133.60	133.66	39.90
14.	80	200	1	91.13	−2.49	131.02	131.04	33.23
15.	80	200	3	87.90	4.18	133.04	133.11	40.88

**Table 4:** Evaluation of Antioxidant Activity and Lycopene in Tomato Peels.

No. crt	Lycopene mg/100 g		DPPH (%)	
	Sunflower Oil	Rapeseed Oil	Sunflower Oil	Rapeseed Oil
1	1.67	1.21	63.58	50.35
2	1.65	1.35	50.66	52.30
3	1.46	1.14	62.66	50.66
4	1.48	1.19	51.79	57.02
5	1.61	0.88	50.76	55.79
6	1.58	0.82	57.43	67.38
7	1.71	0.83	70.46	51.28
8	1.69	1.07	51.28	40.41
9	1.71	1.23	58.05	56.71
10	1.6	1.08	61.53	65.12
11	2.01	0.85	50.66	58.05
12	2.14	1.27	64.61	57.74
13	1.64	1.32	51.89	57.94
14	2	0.9	62.97	61.23
15	1.62	1.01	53.33	60.61

**Table 5:** Refractive index and peroxide value determination in samples extracted with sunflower oil.

No. crt.	Temperature (°C)	Agitation Speed (rpm)	Amount—Tomatoes Pomace (g)	Refractive Index	Peroxide Value meq O_2_/kg
1.	40	150	2	1.4705	0.027
2.	40	250	2	1.4706	0.039
3.	40	200	1	1.4706	0.001
4.	40	200	3	1.4702	1.39
5.	60	150	1	1.4703	1.05
6.	60	150	3	1.4709	1.74
7.	80	150	2	1.4710	1.81
8.	60	200	2	1.4711	1.20
9.	60	200	2	1.4703	1.69
10.	60	200	2	1.4703	1.33
11.	80	250	2	1.4703	1.75
12.	60	150	1	1.4708	1.32
13.	60	250	3	1.4704	1.45
14.	80	200	1	1.4710	1.38
15.	80	200	3	1.4703	1.56

**Table 6:** Refractive index and peroxide value determination in samples extracted with rapeseed oil.

No. crt.	Temperature (°C)	Agitation Speed (rpm)	Amount—Tomatoes Pomace (g)	Refractive Index	Peroxide Value meq O_2_/kg
1.	40	150	2	1.4708	1.20
2.	40	250	2	1.4706	4.13
3.	40	200	1	1.4708	3.25
4.	40	200	3	1.4703	2.43
5.	60	150	1	1.4711	5.64
6.	60	150	3	1.4703	2.55
7.	80	150	2	1.4712	1.21
8.	60	200	2	1.4708	2.41
9.	60	200	2	1.4703	2.01
10.	60	200	2	1.4708	5.72
11.	80	250	2	1.4709	3.12
12.	60	150	1	1.4712	1.80
13.	60	250	3	1.4705	2.46
14.	80	200	1	1.4711	4.45
15.	80	200	3	1.4710	6.13

**Table 7:** Analysis of variance (ANOVA) of the model response for PV content extracted with sunflower oil and rapeseed oil.

Source	PV (meq O_2_/kg)				Lycopene (mg/100 g)				DPPH (%)				Color Difference (ΔE)			
	F-Value *p*-Value		F-Value *p*-Value		F-Value *p*-Value		F-Value *p*-Value		F-Value *p*-Value		F-Value *p*-Value		F-Value *p*-Value		F-Value *p*-Value	
	Sunflower Oil		Rapeseed Oil		Sunflower Oil		Rapeseed Oil		Sunflower Oil		Rapeseed Oil		Sunflower Oil		Rapeseed Oil	
Model	43.49	0.0003	5.19	0.0422	2.94	0.12	2.01	0.0289	6.39	0.0275	3.22	0.0155	2.11	0.2120	11.35	0.0078
X1	0.03	0.8540	0.01	0.9227	0.02	0.88	0.8702	0.3937	19.28	0.0071	0.57	0.4840	0.61	0.4670	34.81	0.0020
X2	93.91	0.0002	1.61	0.2598	1.16	0.33	0.0102	0.9236	1.54	0.2701	1.21	0.3220	2.17	0.2011	3.32	0.1278
X3	3.04	0.1416	11.71	0.0188	9.83	0.02	0.0006	0.9809	4.05	0.1005	9.86	0.0257	1.22	0.3192	2.27	0.1920
X12	114.27	0.0001	6.05	0.0573	0.02	0.87	0.1030	0.7613	0.12	0.7401	0.27	0.6241	0.10	0.7570	11.19	0.0204
X13	141.07	< 0.0001	7.91	0.0374	0.0018	0.96	1.14	0.3337	19.55	0.0069	7.06	0.0450	0.13	0.7328	9.86	0.0256
X23	0.40	0.5506	10.88	0.0215	1.03	0.35	4.13	0.0978	0.27	0.6208	1.06	0.3495	0.01	0.9017	2.26	0.1932
X12	23.31	0.0048	5.16	0.0722	5.25	0.07	1.06	0.3512	0.23	0.6461	8.77	0.0314	4.36	0.0912	11.76	0.0186
X22	5.21	0.0713	3.04	0.1418	1.53	0.27	3.30	0.1291	6.31	0.0538	0.14	0.7233	7.18	0.0438	12.53	0.0166
X32	8.39	0.0339	1.54	0.2703	6.63	0.04	6.78	0.0480	7.37	0.0420	0.01	0.8938	5.46	0.0667	19.88	0.0066
R2	43.49		0.90		0.84		0.78		0.92		0.85		0.79		0.95	
Adj-R2	0.96		0.72		0.55		0.39		0.77		0.58		0.41		0.86	
CV%	3.80		23.58		7.05		12.81		5.11		7.52		43.07		1.34	
Adeq Pre	22.07		7.63		5.20		4.42		9.21		6.77		4.39		9.93	

## Data Availability

The original contributions presented in the study are included in the article, further inquiries can be directed to the corresponding author.
